# Functional disruption of the Golgi apparatus protein ARF1 sensitizes MDA-MB-231 breast cancer cells to the antitumor drugs Actinomycin D and Vinblastine through ERK and AKT signaling

**DOI:** 10.1371/journal.pone.0195401

**Published:** 2018-04-03

**Authors:** Charlotte Luchsinger, Marcelo Aguilar, Patricia V. Burgos, Pamela Ehrenfeld, Gonzalo A. Mardones

**Affiliations:** 1 Department of Physiology, School of Medicine, Universidad Austral de Chile, Valdivia, Chile; 2 Center for Interdisciplinary Studies of the Nervous System (CISNe), Universidad Austral de Chile, Valdivia, Chile; 3 Center for Cell Biology and Biomedicine (CEBICEM), School of Medicine and Science, Universidad San Sebastián, Santiago, Chile; 4 Center for Aging and Regeneration (CARE), Facultad de Ciencias Biológicas, Pontificia Universidad Católica de Chile, Santiago, Chile; 5 Department of Anatomy, Histology and Pathology, School of Medicine, Universidad Austral de Chile, Valdivia, Chile; Institut Jacque Monod, Centre National de la Recherche Scientifique, FRANCE

## Abstract

Increasing evidence indicates that the Golgi apparatus plays active roles in cancer, but a comprehensive understanding of its functions in the oncogenic transformation has not yet emerged. At the same time, the Golgi is becoming well recognized as a hub that integrates its functions of protein and lipid biosynthesis to signal transduction for cell proliferation and migration in cancer cells. Nevertheless, the active function of the Golgi apparatus in cancer cells has not been fully evaluated as a target for combined treatment. Here, we analyzed the effect of perturbing the Golgi apparatus on the sensitivity of the MDA-MB-231 breast cancer cell line to the drugs Actinomycin D and Vinblastine. We disrupted the function of ARF1, a protein necessary for the homeostasis of the Golgi apparatus. We found that the expression of the ARF1-Q71L mutant increased the sensitivity of MDA-MB-231 cells to both Actinomycin D and Vinblastine, resulting in decreased cell proliferation and cell migration, as well as in increased apoptosis. Likewise, the combined treatment of cells with Actinomycin D or Vinblastine and Brefeldin A or Golgicide A, two disrupting agents of the ARF1 function, resulted in similar effects on cell proliferation, cell migration and apoptosis. Interestingly, each combined treatment had distinct effects on ERK1/2 and AKT signaling, as indicated by the decreased levels of either phospho-ERK1/2 or phospho-AKT. Our results suggest that disruption of Golgi function could be used as a strategy for the sensitization of cancer cells to chemotherapy.

## Introduction

In mammalian cells, the Golgi apparatus is organized as a perinuclear compartment arranged in stacked, *cis*- to *trans*-Golgi, membrane-bound cisternae, and a collection of membrane-associated Golgi matrix proteins [1]. The Golgi apparatus is well known as a key compartment at the crossroads of the secretory and endocytic pathways. As such, its function is important not only for the post-translational modification and trafficking of lipids and proteins, but also for the integration of signaling pathways originated at the cell surface [2], or as a hub where distinct signaling pathways originate for the control of cellular processes from within the cell [3]. Hence, the Golgi apparatus is now recognized as a major regulator of cell functions in both normal and transformed cells [4]. Importantly, several unique features of the function of the Golgi apparatus have been found in different types of tumor cells. For instance, a microdeletion found on chromosome 6q21 of the glioblastoma cell line U118MG resulted in the fusion of the gene *FIG*, coding for a Golgi apparatus-associated protein, to the kinase domain of the proto-oncogene c-*ROS* [5]. The resulting protein product FIG-ROS becomes localized to the Golgi apparatus, and this localization leads to constitutive kinase activation and oncogenic transformation [5]. Moreover, some gene products functioning in the Golgi apparatus seem to be essential for different tumor cell types. For example, the silencing of the gene *COPZ1*, which encodes one of two isoforms of the ζ subunit of the coatomer protein complex 1 (COPI), a protein complex involved in vesicle formation at the Golgi apparatus, kills both proliferating and non-dividing tumor cells, including MDA-MB-231 cells, but not normal cells [6]. These findings highlight the central role that the Golgi apparatus plays in tumor cell survival. It is not surprising then that some natural compounds, as well as synthetic compounds, tested initially for their antitumor activity, affect the Golgi apparatus [7–10]. One such natural compound is Brefeldin A (BFA), which, after its discovery as an antiviral and antifungal molecule [11], it was found to have potent antitumor activity [12, 13]. BFA inhibits the activation of the Golgi apparatus regulator ADP-ribosylation factor 1 (ARF1) by ARF guanine nucleotide exchange factors (ARF-GEFs), resulting in disruption of the Golgi apparatus' structure and function, and in inhibition of protein secretion [14]. ARF1 belongs to a family of small GTP-binding proteins that is crucial for eukaryotic cell organization, with functions in vesicular trafficking, lipid homeostasis, and organelle dynamics at both the endoplasmic reticulum-Golgi apparatus and Golgi apparatus-cell surface interfaces [15]. ARF1 is a ubiquitously expressed protein that cycles between cytosolic and membrane-bound pools in a GTP-dependent fashion, and has a prominent role in the recruitment of COPI at the Golgi apparatus [16, 17]. BFA interferes with the initial interaction of ARF1 with membranes [16], resulting in impaired formation of transport vesicles, and thus affecting protein and lipid cargo delivery to different cellular destinations [18]. This effect of BFA on ARF1 has detrimental consequences on several biological processes, including cell proliferation, cell migration, cell invasion, and cell signaling from the cell surface, both in normal and cancer cells [15, 19]. Although the poor bioavailability of BFA precluded its use as anticancer drug [20], a great deal of effort has been placed into the synthesis of BFA analogues and derivatives [21–31], as well as into the finding and development of new ARF1 and ARF-GEF inhibitors [32–35]. This led, for instance, to the identification of Golgicide A (GCA) [36] and the development of LG186 [33], two potent and highly specific inhibitors of GBF1, a *cis*-Golgi ARF-GEF [37]. As a corollary, the Golgi apparatus has been postulated as a suitable target for anti-cancer therapy [38, 39]. Thus, we set to test the hypothesis that treatments that affect the function of the Golgi apparatus sensitize cancer cell lines to conventional antitumor drugs. In this regard, combined drug therapeutic strategies have emerged as potential effective treatments for a variety of cancer types that do not respond well to classic chemotherapy regimens [40–42], including some breast cancer types, such as the so-called triple-negative breast cancer [43]. However, the function of the Golgi apparatus has been the target of few combined treatment surveys [44]. Herein, we show the effect of ARF1 disruptors on the sensitivity of the triple-negative breast cancer cell line MDA-MB-231 to the antitumor drugs Actinomycin D (ActD) and Vinblastine (VLB). ActD inhibits transcription and replication by binding to selected sites in single-stranded DNA [45], double-stranded DNA [46], or quadruplex DNA [47]. ActD also affects mitosis by reducing the binding of the mitotic regulator RCC1 to chromosomes, which leads to spindle defects and cell death by mitotic catastrophe [48]. ActD is used in several combined drug treatments [49–51], but it is typically not employed against breast cancer. VLB, on the other hand, suppresses microtubule dynamics, leading to mitotic block and apoptosis [52]. Because dynamic, mitotic spindle microtubules are among the most successful targets for anticancer therapy [53], vinblastine is frequently used in combined drug treatments [54–57], including for breast cancer [58]. In our present report, we show the unexpected result that the combined use of ARF1 disruptors and ActD or VLB acted synergistically on MDA-MB-231 cells.

## Materials and methods

### Cell culture

MDA-MB-231 (human breast adenocarcinoma) cells were obtained from the American Type Culture Collection (Manassas, VA), and were maintained in DMEM-F12 medium supplemented with 10% heat-inactivated fetal bovine serum, 100 U/ml penicillin, 100 μg/ml streptomycin (Life Technologies), and 5 μg/ml plasmocin (InvivoGen, San Diego, CA), in a humidified incubator with 5% CO_2_ at 37°C.

### Antibodies and cell reagents

We used the following mouse monoclonal antibodies: clone E10 to phospho-p44/42 MAPK (ERK1/2) (Thr202/Tyr204) (Cell Signaling), clone 16B12 to Influenza Hemagglutinin (HA) epitope (Abcam), and clone 35/GM130 to GM130 (BD Biosciences). We used the following rabbit monoclonal antibodies: clone C67E7 to AKT (Cell Signaling), clone D9E to phospho-AKT (Ser473; Cell Signaling), and clone 269518 to cleaved Caspase-3 (R&D Systems). We used polyclonal antibodies to the following proteins: p44/42 MAPK (ERK1/2) (cat # 9102, Cell Signaling), Caspase-3 (cat # 9662, Cell Signaling), Giantin (cat # NBP2-22321, Novus Biologicals), and TGN46 (cat # AHP500G, AbD Serotec). The following fluorochrome-conjugated antibodies were from Life Technologies: Alexa Fluor-594–conjugated donkey anti mouse IgG, Alexa Fluor-647-conjugated donkey anti mouse IgG, Alexa Fluor-488–conjugated donkey anti rabbit IgG, and Alexa Fluor-647-conjugated donkey anti sheep IgG. HRP-conjugated secondary antibodies were from Jackson ImmunoResearch. Primary antibodies were used at a dilution 1/200 to 1/2000. HRP- or Alexa Fluor-conjugated secondary antibodies were used at dilutions 1/1000 to 1/20000, depending on their reactivity. Actinomycin D (ActD), Brefeldin A (BFA), Golgicide A (GCA), Vinblastine (VLB), and a cocktail of protease inhibitors were from Sigma-Aldrich (St. Louis, MO). The fluorescent nuclear stain 4’,6-diamidino-2-phenylindole (DAPI) was from Life Technologies. Plasmids encoding HA-epitope-tagged ARF1 variants (HA-ARF1-T31N and HA-ARF1-Q71L) were kindly provided by J. Bonifacino (NICHD, NIH, USA), and were described elsewhere [59].

### Transient transfection and immunofluorescence microscopy

Transient transfections to express HA-epitope-tagged ARF1 variants were performed using Lipofectamine 2000 (Life Technologies), according to the manufacturer's instructions. After 3, 8 or up to 16-h, transfected cells were left untreated for further 60 min or treated for 60 min either with 10 ng/ml ActD or 25 nM VLB. Alternatively, cells were treated for 60 min either with 5 μg/ml BFA, 10 μM GCA, 10 ng/ml ActD or 25 nM VLB, or treated for 60 min either with 5 μg/ml BFA or 10 μM GCA in conjunction either with 10 ng/ml ActD or 25 nM VLB. Cells were processed by immunofluorescence microscopy as described previously [60], which included fixing cells in methanol or 4% paraformaldehyde depending on primary antibody reactivity. Fluorescence microscopy images were acquired with an AxioObserver.D1 microscope equipped with a PlanApo 63x oil immersion objective (NA 1.4), and an AxioCam MRm digital camera (Carl Zeiss), using similar settings as described previously [60]. To prepare figures, images were processed with Image J software (version 1.44o; Wayne Rasband, NIH, http://imagej.nih.gov) and Adobe Photoshop CS3 software (Adobe Systems, Mountain View, CA).

### Cell proliferation

For cell proliferation assays, 5 x 10^3^ cells were seeded in 96-well plates and incubated for 16 h at 37°C in starvation medium (medium without fetal bovine serum). Cells were then left untreated for further 3-h, or subjected for 3-h to transfection with increasing concentrations (0.4–10 μg/ml) of the plasmid encoding either of the HA-epitope-tagged ARF1 variants. Cells were further incubated for 24-h at 37°C in starvation medium supplemented with 0.5 μCi/ml [^3^H]-thymidine (PerkinElmer), or, in the case of cells transfected with 0.4 μg/ml of plasmid DNA, incubated in the presence of either 10 ng/ml ActD or 25 nM VLB. Alternatively, cells were incubated for 16 h at 37°C in starvation medium, followed by incubation for 24-h at 37°C in starvation medium supplemented with 0.5 μCi/ml [^3^H]-thymidine in the presence of increasing concentrations of either BFA (0.2–5 μg/ml), GCA (0.4–10 μM), ActD (0.4–10 ng/ml) or VLB (1–10 nM), or either with 5 μg/ml BFA or 10 μM GCA in conjunction either with 10 ng/ml ActD or 25 nM VLB. After removing the labeling medium, cells were washed with cold PBS, subjected to trypsinization, and collected onto Whatman Grade GF/A glass microfiber filters (GE Healthcare) using a Multimash 2000 cell harvester (Dynatech). After addition of Ecoscient^TM^ scintillation liquid (National Diagnostics), the radioactivity on the filters was determined by a Tri-carb 2100tr liquid scintillation analyzer (Packard).

### Wound healing assay

For two-dimensional, wound-healing assays, 2.5 x 10^4^ cells were seeded in 12-well plates and incubated at 37°C in complete medium. After 16-h, semi-confluent cells were left untreated or subjected to transfection with 0.4 μg/ml of the plasmid encoding either of the HA-epitope-tagged ARF1 variants. After additional 16-h, confluent cells were wounded with a sterile tip, and after washing cell debris with PBS, three phase-contrast images of different regions of the wounds were acquired with an AxioObserver.D1 microscope equipped with an A-Plan 5x objective (NA 0.12), and an AxioCam MRm digital camera (Carl Zeiss). Wounded, non-transfected cells were left untreated or treated either with 5 μg/ml BFA, 10 μM GCA, 10 ng/ml ActD or 25 nM VLB, or either with 5 μg/ml BFA or 10 μM GCA in conjunction either with 10 ng/ml ActD or 25 nM VLB. Wounded, transfected cells were left untreated or treated either with 10 ng/ml ActD or 25 nM VLB. After 20-h, images of the same regions were acquired, and the area of wound closure was quantified using Image J software (version 1.44o).

### Preparation of protein extracts, protein electrophoresis and immunoblotting

Protein extract preparation, SDS-PAGE analysis and immunoblotting were performed using methods that we have described previously [60–62].

### Apoptosis analyses

We used three methods to assess the apoptotic state of MDA-MB-231 cells: 1) Binding of Alexa Fluor-488–conjugated Annexin V (Life Technologies), 2) Immunofluorescence with antibody to cleaved Caspase-3, and 3) Immunoblotting to Caspase-3. For Annexin V binding, 2.5 x 10^4^ cells were seeded on 12-mm round, glass coverslips in a 24-well plate and maintained in complete culture medium. After 24-h, cells were left untreated for further 11-h or subjected for 11-h either to single treatments or to the combined treatment of ARF1 disruptors (0.4 μg/ml HA-epitope-tagged ARF1 variants, 5 μg/ml BFA or 10 μM GCA) and the antitumor drugs (10 ng/ml ActD or 25 nM VLB). Cells were stained with Alexa Fluor-488–conjugated Annexin V and propidium iodide using the Dead Cell Apoptosis Kit, according to the manufacturer's instructions (Thermo Fisher Scientific), and fixed in 4% paraformaldehyde for 30 min at room temperature. After washing in PBS, coverslips were mounted on microscope glass slides using Fluoromount-G mounting media (Thermo Fisher Scientific). Fluorescence microscopy images of ten, random fields were acquired for each treatment with an AxioObserver.D1 microscope equipped with a LD A-Plan 40x objective (NA 0.5), and an AxioCam MRm digital camera (Carl Zeiss). The number of early apoptotic versus late apoptotic/necrotic cells was quantified considering Annexin V-only staining and Annexin V plus propidium iodide staining, respectively. Only early apoptotic cells were compared among the different treatments. For the immunofluorescence with antibody to cleaved Caspase-3, 2.5 x 10^4^ cells were seeded on 12-mm round, glass coverslips in a 24-well plate, subjected to the same treatments indicated above, and processed for immunofluorescence as described above. Fluorescence microscopy images of ten, random fields were acquired for each treatment as indicated above, and the number of cells with activated Caspase-3 staining (regarded as apoptotic cells) was quantified. For the immunoblotting to Caspase-3, 1 x 10^5^ cells were seeded on each well of 6-well plates. Confluent cells were incubated in starvation medium for 16-h at 37°C, followed by the same treatments indicated above, but for 5-h at 37°C, and processed for immunobloting as cited above. The levels of Caspase-3 and cleaved Caspase-3 were estimated by densitometry analysis of the immunoblot signal (see below) and compared among the different treatments.

### Densitometry quantification and statistical analysis

The amount of immunoblot signal from images with unsaturated pixels was estimated using Image J software (version 1.44o). For each condition, protein bands were quantified from at least three independent experiments. Statistical analysis was performed using Microsoft Excel for Mac 2011 (Microsoft Corporation). When appropriate, results were represented in graphs depicting the mean ± standard deviation. Statistical significance was determined by two-tailed, paired *t*-test. *P*-values > 0.05 or *≤* 0.05 were regarded as not statistically significant or statistically significant, respectively. In the figures, *P*-values between 0.01 and 0.05 are indicated with one asterisk, *P*-values between 0.001 and 0.01 are indicated with two asterisks, and *P*-values less than 0.001 are indicated with three asterisks.

## Results and discussion

### The Golgi apparatus of MDA-MB-231 cells is sensitive to ARF1 disruptors

**Fig 1:**
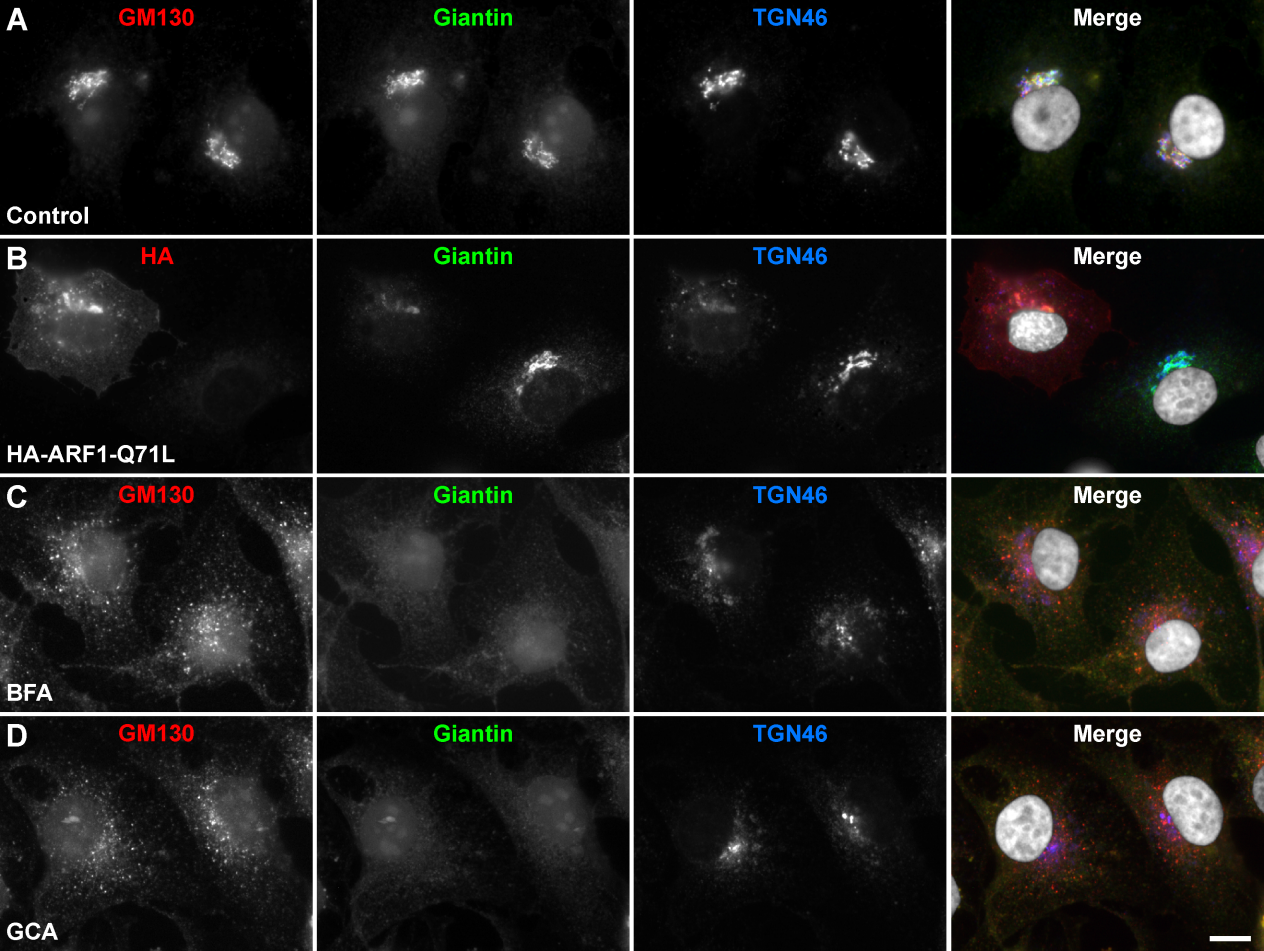
Effect of Golgi disrupting treatments on the Golgi apparatus of MDA-MB-231 cells. Cells were left untreated (A; *Control*), or transfected to transiently express the HA-epitope-tagged ARF1 constitutively-activated mutant for 16 h (B; *HA-ARF1-Q71L*), or treated for 60 min either with 5 μg/ml Brefeldin A (C; *BFA*) or 10 μM Golgicide A (D; *GCA*). Cells were fixed, permeabilized, and immunolabeled with mouse monoclonal antibody to GM130, rabbit polyclonal antibody to Giantin, and sheep antibody to TGN46. Secondary antibodies were Alexa-594-conjugated donkey anti-mouse IgG (red channel), Alexa-488-conjugated donkey anti-rabbit IgG (green channel), and Alexa-647-conjugated donkey anti-sheep IgG (blue channel). Nuclei were stained with DAPI (gray channel). Stained cells were examined by fluorescence microscopy. Merging red, green, blue, and grey channels generated the fourth image on each row; yellow indicates overlapping localization of the red and green channels, cyan indicates overlapping localization of the green and blue channels, magenta indicates overlapping localization of the red and blue channels, and white indicates overlapping localization of all three channels. Bar, 10 μm.

Because the Golgi apparatus of different cell lines may respond differently to treatments expected to affect its function, first we determined whether ARF1 disruptors had the expected effects on the structural organization of the Golgi apparatus of MDA-MB-231 cells. We either transfected cells to transiently express HA-epitope-tagged ARF1 variants, or treated cells with BFA or GCA, followed by immunofluorescence microscopy analysis. We chose to express the ARF1 variants T31N or Q71L on the basis of their functional properties. ARF1-T31N has very low affinity for GTP, and behaves as a dominant negative mutant [63], and ARF1-Q71L has drastically reduced GTPase activity, and behaves as a constitutively activated mutant [64]. The immunofluorescence analysis of untreated cells showed perinuclear colocalization of the *cis*-Golgi matrix protein GM130, the *cis*-Golgi protein Giantin, and the *trans*-Golgi protein TGN46 (Fig 1A), indicating that this cell line exhibits a typical Golgi apparatus architecture [65]. In contrast, expression of ARF1-Q71L resulted in distribution of this set of proteins in both the Golgi apparatus and peripheral puncta (Fig 1B, and data not shown). These localizations were expected, however, because expression of ARF1-Q71L produces some degree of halt of vesicular transport in pre-Golgi compartments [63, 64]. Expression of ARF1-T31N (data not shown), as well as the treatment either with BFA (Fig 1C) or GCA (Fig 1D), also resulted in redistribution of these proteins: GM130 to scattered cytoplasmic puncta (Fig 1C and 1D, and data not shown), Giantin to the endoplasmic reticulum (Fig 1C and 1D, and data not shown), and TGN46 to the centrosome (Fig 1C and 1D, and data not shown). Again, these changes in localization were expected, because the disruption of ARF1 function results in Golgi matrix proteins being redistributed to puncta near endoplasmic reticulum exit sites, of *cis*-Golgi proteins to the endoplasmic reticulum, and of *trans*-Golgi proteins to the centrosome [33, 36, 63, 66–69]. Thus, all these observations indicate that these treatments could be used to disrupt the Golgi apparatus of MDA-MB-231 cells. In addition, the effects of these treatments are also in agreement to previous reports demonstrating that the knockdown of ARF1 is deleterious to MDA-MB-231 cells [70–72].

### The Golgi apparatus of MDA-MB-231 cells is sensitive to VLB

**Fig 2:**
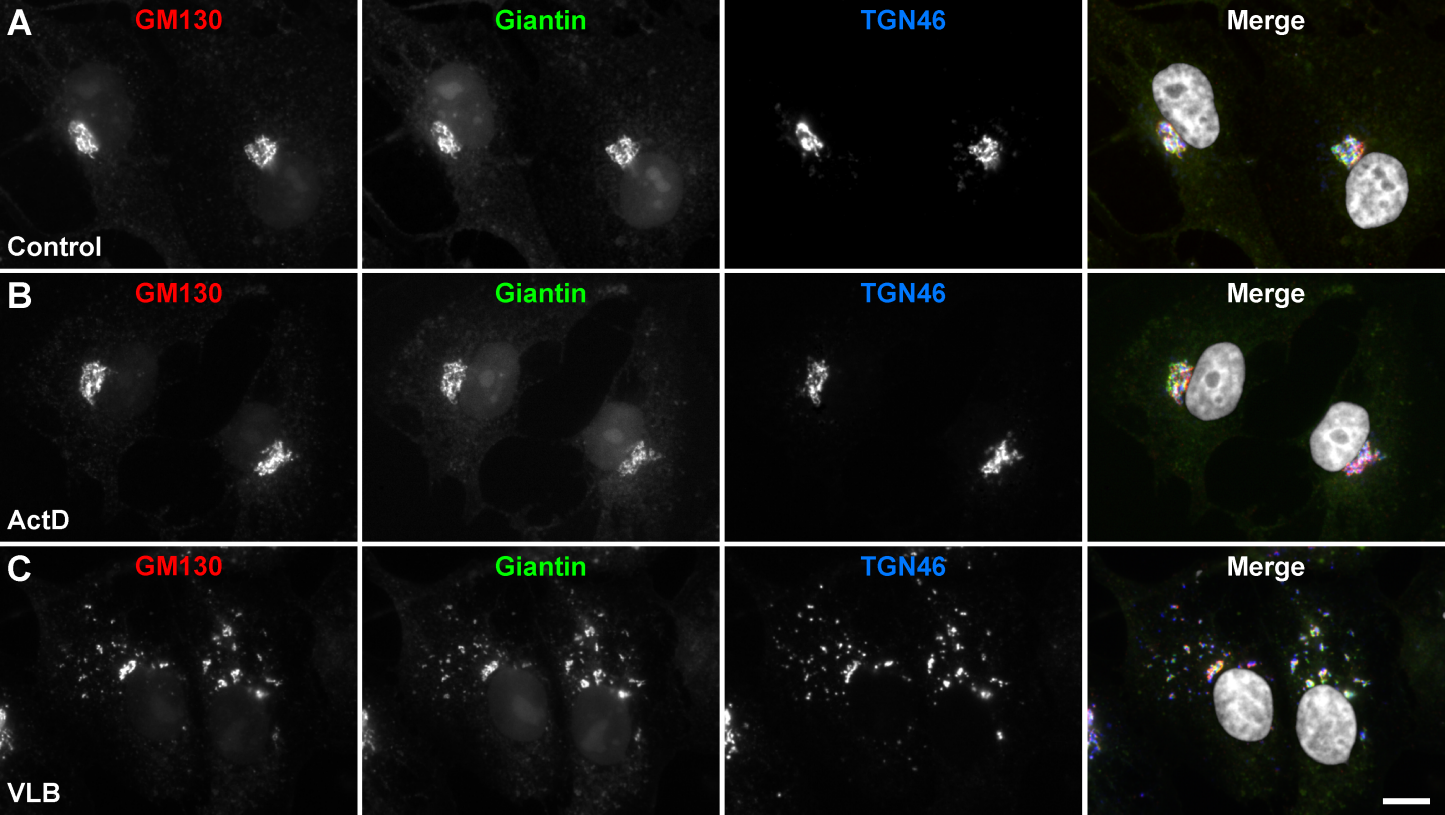
Effect of Actinomycin D and Vinblastine on the Golgi apparatus of MDA-MB-231 cells. Cells were left untreated (A; *Control*), or treated for 60 min either with 10 ng/ml Actinomycin D (B; *ActD*) or 25 nM Vinblastine (C; *VLB*). Cells were fixed, permeabilized, and immunolabeled with mouse monoclonal antibody to GM130, rabbit polyclonal antibody to Giantin, and sheep antibody to TGN46. Secondary antibodies were Alexa-594-conjugated donkey anti-mouse IgG (red channel), Alexa-488-conjugated donkey anti-rabbit IgG (green channel), and Alexa-647-conjugated donkey anti-sheep IgG (blue channel). Nuclei were stained with DAPI (gray channel). Stained cells were examined by fluorescence microscopy. Merging red, green, blue, and grey channels generated the fourth image on each row; yellow indicates overlapping localization of the red and green channels, cyan indicates overlapping localization of the green and blue channels, magenta indicates overlapping localization of the red and blue channels, and white indicates overlapping localization of all three channels. Bar, 10 μm.

Before testing whether the disruption of the Golgi apparatus sensitizes MDA-MB-231 cells to the antitumor drugs ActD and VLB, we evaluated by fluorescence microscopy the effect of the treatment with each antitumor drug alone on the integrity of the Golgi apparatus. We chose ActD because it is one of the oldest chemotherapy drugs used to treat different types of cancer [73]. ActD is a polypeptide antibiotic isolated from *Streptomyces* bacteria, and was the first antibiotic used for the treatment of a variety of cancers that include Ewing’s sarcoma, gestational trophoblastic cancer, rhabdomyosarcoma, testicular cancer, and Wilms’s tumor [74]. VLB, on the other hand, is an alkaloid isolated from the periwinkle plant *Catharanthus roseus*, used for the treatment of several types of cancer, including Hodgkin’s lymphoma [56], non-small cell lung cancer [55], bladder cancer [57], and melanoma [54]. It has also been shown to be effective on breast cancer cell lines including MDA-MB-231 cells [58, 75]. We observed that ActD produced no noticeable effect on the perinuclear localization of the Golgi apparatus, assessed by immunofluorescence to Giantin, GM130 and TGN46 (Fig 2A and 2B). It is known that the main action of ActD is via its inhibition of transcription [76], therefore a direct effect on Golgi apparatus structural organization was not expected. In contrast, treatment with VLB resulted in dispersal of Golgi elements throughout the cytoplasm of MDA-MB-231 cells (Fig 2C), in agreement with earlier reports showing a similar effect in other cell lines [77–79]. Moreover, the scattered fragments showed colocalization of the three proteins (Fig 2C), indicating that they corresponded to bona fide Golgi stacks. However, Golgi stacks dispersion was an expected effect of the treatment with VLB. This is because VLB binds to the cytoplasmic protein tubulin inhibiting the assembly of microtubules [52], and the architecture of the Golgi apparatus depends on microtubule assembly integrity [80]. On the other hand, treatment with either antitumor drug, in conjunction with either ARF1 disruptor, did not prevent or alter the effect observed on the Golgi apparatus when cells were treated with each of the ARF1 disruptors alone (S1 Fig).

### The treatment of MDA-MB-231 cells with ActD or VLB in conjunction with ARF1 disruptors produces a synergistic reduction in cell proliferation

**Fig 3:**
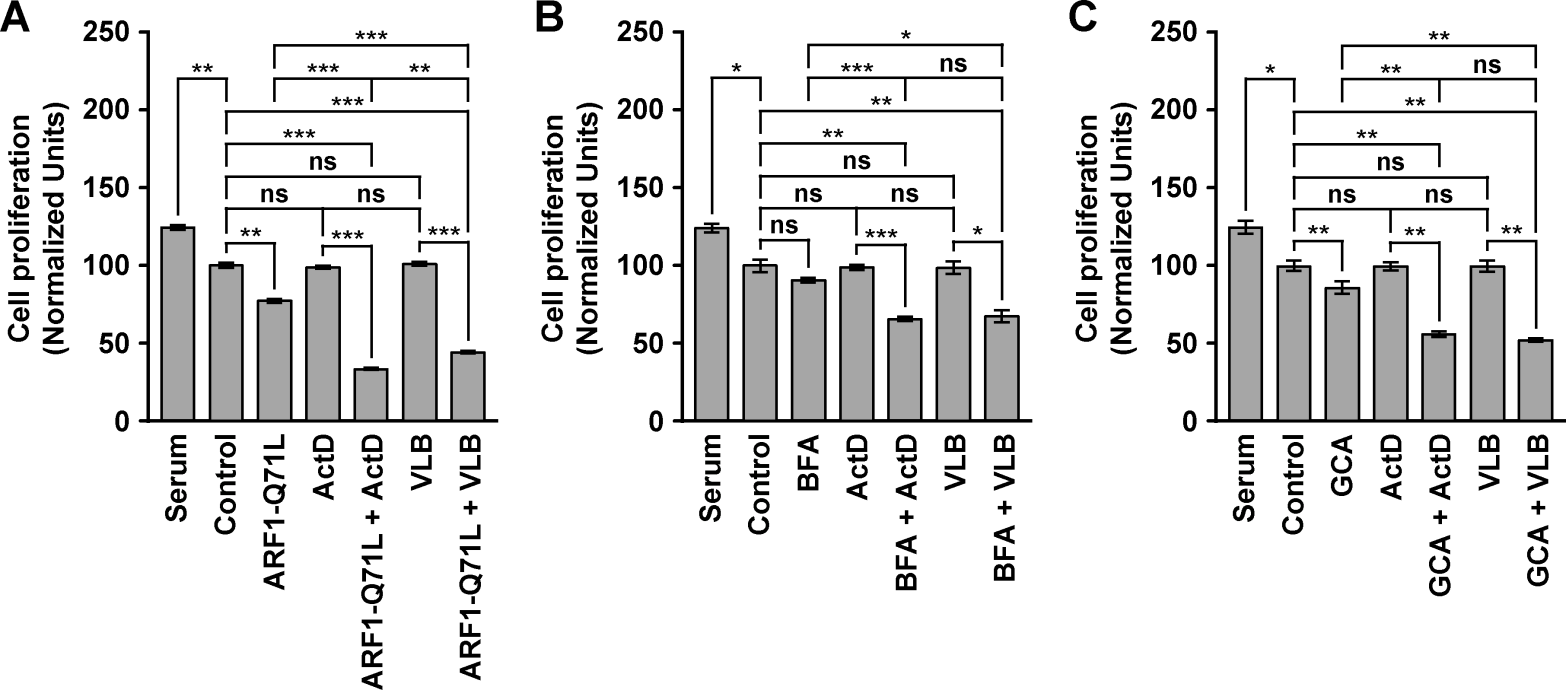
Effect of the combined treatment with Golgi disrupting agents and Actinomycin D or Vinblastine on the proliferation of MDA-MB-231 cells. (A) Cells were left in normal culture medium containing 10% FBS, or transfected to transiently express the HA-epitope-tagged ARF1 constitutively-activated mutant (*ARF1-Q71L*) for 16 h. Untransfected cells were either maintained in normal culture medium containing 10% FBS for 24 h (*Serum*), or serum-starved and either left untreated for additional 24 h (*Control*) or treated 24 h either with 0.4 ng/ml Actinomycin D (*ActD*) or 1 nM Vinblastine (*VLB*). Transfected cells were serum-starved, and either left without further treatment for additional 24 h (*ARF1-Q71L*), or treated either with ActD (*ARF1-Q71L + ActD*) or VLB (*ARF1-Q71L + VLB*), as in untransfected cells. (B) Untransfected cells were treated as in *A*, or serum-starved and treated for 24 h either with 0.2 μg/ml Brefeldin A alone (*BFA*), or in conjunction with ActD (*BFA + ActD*) or VLB (*BFA + VLB*). (C) Untransfected cells were treated as in *A*, or serum-starved and treated for 24 h either with 2 μM Golgicide A alone (*GCA*), or in conjunction with ActD (*GCA + ActD*) or VLB (*GCA + VLB*). In all conditions, cells were cultured during the last 24 h in the presence of [^3^H]-thymidine. Cells were harvested, and [^3^H]-thymidine incorporation was quantified with a scintillation counter. Bar represents the mean ± standard deviation (n = 3). * *P* < 0.05; ** *P* < 0.01; *** *P* < 0.001; ns, not statistically significant.

To test whether ARF1 disruptors sensitize MDA-MB-231 cells to ActD or VLB, we first evaluated cell proliferation. As expected [75, 81], we found that the treatment with either of the antitumor drugs alone produced a significant, dose-dependent decrease in cell proliferation (Graphs A and B in S2 Fig). We also found that each of the ARF1 disrupting treatments produced a significant, dose-dependent decrease in cell proliferation (Graphs C-E in S2 Fig, and data not shown). The effects of ARF1-T31N, BFA and GCA are in agreement with the effects already reported of BFA and ARF1-T31N in MDA-MB-231 cells [71, 82]. In contrast, the effect of ARF1-Q71L was unexpected, as it has been established that overexpression of endogenous ARF1 correlates with increased cell proliferation in different tumor types [83–86]. Moreover, expression of ARF1-Q71L also has been correlated to increased cell proliferation in several cancer cell lines, including MDA-MB-231 cells [71]. This apparent discrepancy of our results could be due to different experimental setups: while the majority of reports evaluating the effect of ARF1-Q71L expression have analyzed cell proliferation during several days, we analyzed cell proliferation during 24-h. Thus, it is plausible that the expression of ARF1-Q71L during a short period of time (i.e., 24-h) could result in initial impairment of mitosis. In this regard, it has been shown that both AMP-activated protein kinase and cyclin-dependent kinase 1 phosphorylate GBF1 during mitosis inhibiting its activity, which results in disassembly of the Golgi apparatus [87, 88]. Because different steps of Golgi apparatus disassembly seem to control mitotic entry and progression [89], it is possible that ARF1-Q71L could initially overcome some of these regulatory mechanisms. On the other hand, we found that the decrease in cell proliferation was greater in cells expressing ARF1-Q71L compared to cells treated with BFA or GCA alone (Fig 3A–3C). This could be due to early effects of ARF1-Q71L expression, as mentioned before, and/or to the relatively high level of expression of ARF1 in MDA-MB-231 cells [70, 72], which could render these cells less sensitive to BFA and GCA. Surprisingly, compared to single treatments, each of the ARF1 disrupting treatments combined with any of the antitumor drugs resulted in a significant, greater decrease in cell proliferation than expected for an additive effect (Fig 3A–3C), even in combinations at relatively low doses (our unpublished results). More specifically, the expression of ARF1-Q71L resulted in a significant reduction on the levels of cell proliferation to 77.4 ± 1.3%, compared to the levels of untreated cells (Fig 3A, *Control* versus *ARF1-Q71L*). Conversely, treatment with 0.4 ng/ml ActD or 1 nM VLB resulted in effects on cell proliferation that were not significant when compared to the levels of untreated cells (Fig 3A–3C, *Control* versus *ActD*, and *Control* versus *VLB*). In contrast, when compared to untreated cells, ARF1-Q71L expression combined with ActD or VLB resulted in significant reductions in cell proliferation to 33.6 ± 0.7% (Fig 3A, *Control* versus *ARF1-Q71L + ActD*) and 44.3 ± 0.9% (Fig 3A, *Control* versus *ARF1-Q71L + VLB*), respectively, indicating that the combined treatments acted synergistically. Likewise, although the treatment with 0.2 μg/ml BFA resulted in a reduction of cell proliferation that reached only 91.0 ± 1.5% of the levels found in untreated cells (Fig 3B, *Control* versus *BFA*), the combined treatment with each of the antitumor drugs resulted in reductions that are consistent with a synergistic effect: to 65.7 ± 1.6% for the combination with ActD (Fig 3B, *Control* versus *BFA + ActD*), and to 67.5 ± 4.0% for the combination with VLB (Fig 3B, *Control* versus *BFA + VLB*). We found similar synergistic reductions of cell proliferation when cells where subjected to the combined treatments with GCA: while the treatment with 2 μM GCA alone resulted in a significant reduction of cell proliferation to 86.0 ± 4.1%, compared to the levels of untreated cells (Fig 3C, *Control* versus *GCA*), the combined treatment either with ActD or VLB resulted in significant reductions to 56.0 ± 2.0% (Fig 3C, *Control* versus *GCA + ActD*) and 52.1 ± 1.1% (Fig 3C, *Control* versus *GCA + VLB*), respectively.

### The treatment of MDA-MB-231 cells with ActD or VLB in conjunction with ARF1 disruptors produces a synergistic reduction in cell migration

**Fig 4:**
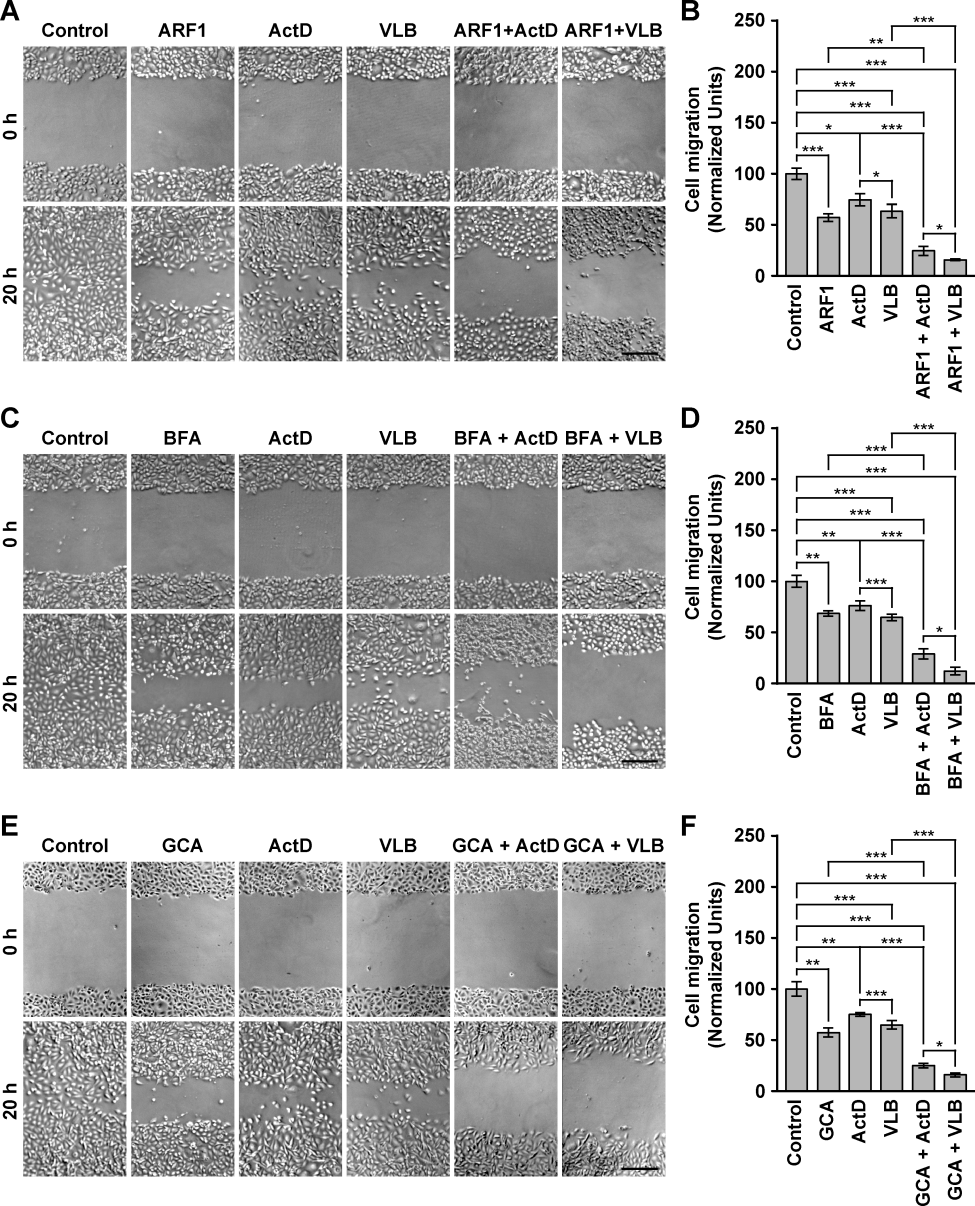
Effect of the combined treatment with Golgi disrupting agents and Actinomycin D or Vinblastine on the migration of MDA-MB-231 cells. (A) Cells were left untreated, or transfected to transiently express the HA-epitope-tagged ARF1 constitutively-activated mutant (*ARF1*) for 16 h. Cultures of confluent cells were wounded with a sterile tip, cells were serum-starved, and either left untreated for additional 20 h (*Control* and *ARF1*), or treated 20 h either with 10 ng/ml Actinomycin D (*ActD* and *ARF1 + ActD*) or 25 nM Vinblastine (*VLB* and *ARF1 + VLB*). (B) Cultures of confluent cells were wounded as in *A*, cells were serum-starved, and either left untreated for additional 20 h (*Control*), or treated 20 h either with 5 μg/ml Brefeldin A (*BFA*), 10 ng/ml Actinomycin D (*ActD*) or 25 nM Vinblastine (*VLB*), or with BFA in conjunction either with ActD (*BFA + ActD*) or VLB (*BFA + VLB*). (C) Cultures of confluent cells were wounded as in *A*, cells were serum-starved, and either left untreated for additional 20 h (*Control*), or treated 20 h either with 10 μM Golgicide A (*GCA*), 10 ng/ml Actinomycin D (*ActD*) or 25 nM Vinblastine (*VLB*), or with GCA in conjunction either with ActD (*GCA + ActD*) or VLB (*GCA + VLB*). Images of the same regions were taken immediately after the wounding (*0 h*), and after 20-h of treatment (*20 h*). (B, D and F) Cell migration, under the treatments shown in *A*, *C* and *E*, was estimated as the area re-occupied by cells after the 20-h treatment. Bar represents the mean ± standard deviation (n = 3). * *P* < 0.05; ** *P* < 0.01; *** *P* < 0.001. Bar, 200 μm.

MDA-MB-231 cells are widely used as an experimental model of human breast cancer metastasis [90]. Therefore, we next evaluated cell migration by a wound-healing assay. Cells were either transfected with ARF1 constructs or left untreated until they were confluent. The confluent monolayer of cells was wounded, and cells were either left with no further treatment, treated with each antitumor drug alone, treated with each ARF1 disruptor alone, or treated with each antitumor drug in conjunction with each ARF1 disruptor. The progression of the wound closure was monitored by light microscopy, collecting images at the beginning and 20-h after the beginning of the treatments. We found that untreated cells occupied the area of the wound almost completely after 20 h (Fig 4). In contrast, significantly fewer cells were present in the wounds of cells subjected to any of the single treatments (Fig 4), indicating impaired cell migration. Similar impairment on cell migration has been reported for MDA-MB-231 cells treated either with BFA [82] or ActD [91]. The effects of the single treatments with the ARF1 disruptors are consistent with the role of ARF1 in regulating cell migration in MDA-MB-231 cells by controlling both Rac1, a Rho GTPase associated with lamellipodia formation during cell migration [92], and the formation of focal adhesions [93]. Importantly, each of the combined treatments resulted in a decrease in cell migration in a magnitude consistent with a synergistic effect (Fig 4).

### The treatment of MDA-MB-231 cells with ActD or VLB in conjunction with ARF1 disruptors produces a synergistic increase in apoptosis

**Fig 5:**
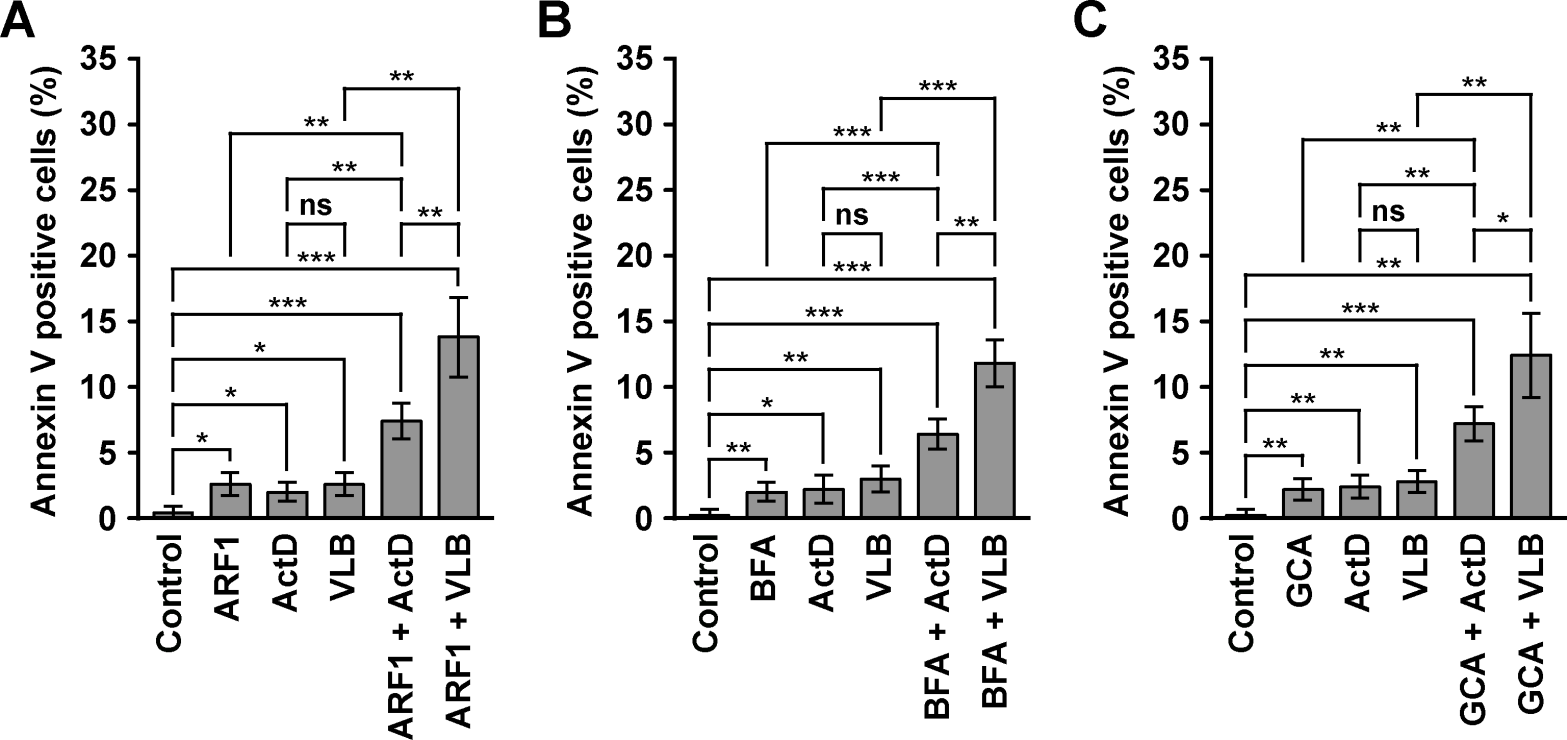
Effect of the combined treatment with Golgi disrupting agents and Actinomycin D or Vinblastine on the apoptosis of MDA-MB-231 cells. (A) Cells were left untreated, or transfected to transiently express the HA-epitope-tagged ARF1 constitutively-activated mutant (*ARF1*) for 16 h. Untransfected cells were left untreated for further 12 h (*Control*), or treated 12 h either with 10 ng/ml Actinomycin D (*ActD*) or 25 nM Vinblastine (*VLB*). Transfected cells were left untreated for further 12 h (*ARF1*), or treated 12 h either with 10 ng/ml Actinomycin D (*ARF1 + ActD*) or 25 nM Vinblastine (*ARF1 + VLB*). (B) Cells were left untreated for 12 h (*Control*), or treated 12 h either with 5 μg/ml Brefeldin A (*BFA*), 10 ng/ml Actinomycin D (*ActD*) or 25 nM Vinblastine (*VLB*), or with BFA in conjunction either with ActD (*BFA + ActD*) or VLB (*BFA + VLB*). (C) Cells were left untreated for 12 h (*Control*), or treated 12 h either with 10 μM Golgicide A (*GCA*), 10 ng/ml Actinomycin D (*ActD*) or 25 nM Vinblastine (*VLB*), or with GCA in conjunction either with ActD (*GCA + ActD*) or VLB (*GCA + VLB*). (A-C) Graphs depict the quantification of the number of cells decorated with Alexa-488-conjugated Annexin V. Bar represents the mean ± standard deviation (n = 3). * *P* < 0.05; ** *P* < 0.01; *** *P* < 0.001; ns, not statistically significant.

To further analyze the sensitivity of MDA-MB-231 cells to the combined treatments of ActD or VLB and ARF1 disruptors, we analyzed cell death by apoptosis, by assessing binding of cells to Alexa-Fluor-488-conjugated Annexin V. Both ActD and VLB induce cell death by apoptosis [94, 95], and accordingly we found that both significantly increased the apoptosis of MDA-MB-231 cells (Fig 5). We also found that the treatment with each of the ARF1 disruptors significantly increased the apoptosis of MDA-MB-231 cells (Fig 5, and data not shown), in agreement with previous reports [69, 96]. Importantly, the combined treatments also resulted in significant increases in apoptosis, but to a higher extent than in single ARF1 disruptor treatments, or single antitumor drug treatments (Fig 5). Thus, the magnitude of the increases in apoptosis observed with combined treatments was indicative of synergistic effects (Fig 5), which is consistent with the effects on cell proliferation (Fig 3) and cell migration (Fig 4). Interestingly, the combined treatment of any of the ARF1 disruptors with VLB resulted in significantly higher increases in apoptosis compared to the combined treatments with ActD, although the difference in the increase of apoptosis in cells treated with VLB or ActD alone was not significant (Fig 5). We obtained similar results in experiments assessing apoptosis either by immunofluorescence or immunoblot to cleaved Caspase-3 (our unpublished results). Together, these results indicate that MDA-MB-231 cells are more prone to apoptosis with the combined treatments of ARF1 disruptors and either of the antitumor drugs.

### The treatment of MDA-MB-231 cells with ActD or VLB in conjunction with ARF1 mutants or BFA affects ERK1/2 signaling

**Fig 6:**
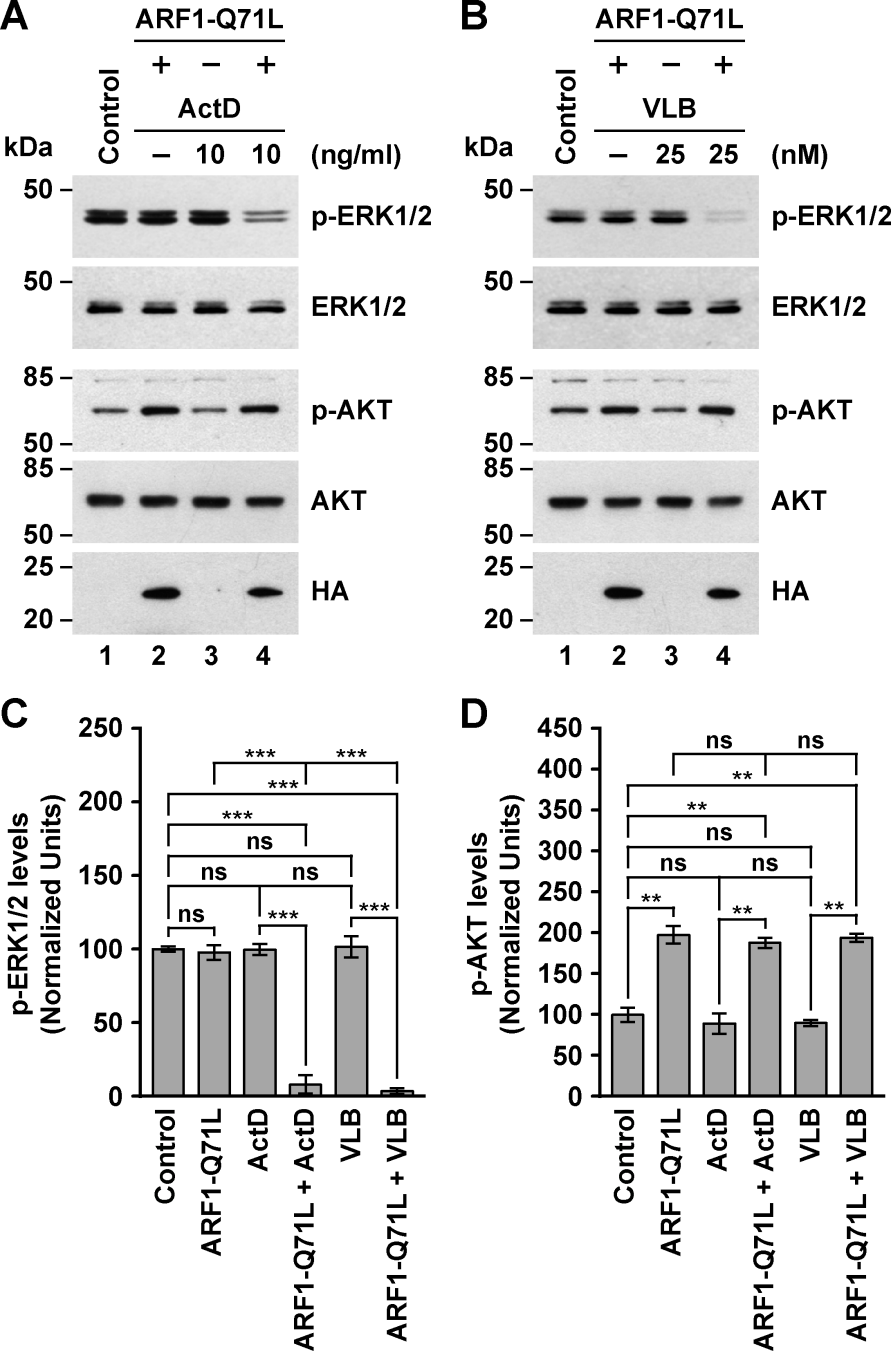
Effect of the expression of the constitutively-activated ARF1 mutant in conjunction with the treatment with Actinomycin D or Vinblastine on the levels of phospho-ERK1/2 and phospho-AKT in MDA-MB-231 cells. (A-B) Cells were left untreated (*Control*), or transfected to transiently express for 16 h the HA-epitope-tagged constitutively-activated ARF1 mutant (*ARF1-Q71L*). Cells were left untreated for further 5 h (*Control* and *ARF1-Q71L*), or treated 5 h either with 10 ng/ml Actinomycin D (*ActD*; A) or 25 nM Vinblastine (*VLB*; B). After solubilizing in detergent, proteins were analyzed by SDS-PAGE followed by immunoblotting using antibodies to the proteins indicated on the right, or to the HA-epitope to detect HA-epitope-tagged ARF1 mutant. The position of molecular mass markers is indicated on the left. (C-D) Densitometry quantification of the immunoblot signal of the levels of phospho-ERK1/2 as shown in *A* and *B* (C), and of the levels of phospho-AKT as shown in *A* and *B* (D). Bar represents the mean ± standard deviation (n = 3). ** *P* < 0.01; *** *P* < 0.001; ns, not statistically significant.

Several reports indicate that the MAPK/ERK1/2 signaling pathway is frequently abnormally activated in breast cancer [97]. Similarly, the PI3K/AKT signaling pathway plays important roles in both estrogen receptor negative and estrogen receptor positive breast tumor cells [97, 98]. In fact, both pathways seem to mediate several tumorigenic responses in MDA-MB-231 cells [99], and in an ARF1-dependent manner [96]. Thus, to explore the mechanism of sensitization of MDA-MB-231 cells to ActD and VLB, we analyzed the ERK1/2 and AKT signaling pathways evaluating the levels of phospho-ERK1/2 and phospho-AKT by immunoblot. We found that the expression of ARF1 variants did not change the levels of phospho-ERK1/2 when compared to mock transfected cells (Lanes 1 and 2 in Fig 6A and 6B, Fig 6C, images A and B in S3 Fig, and graph C in S3 Fig). This was surprising considering that ARF1 overexpression, as well as ARF1-Q71L expression, results in increased levels of phospho-ERK1/2 in other cell lines [100]. One possibility is that the high levels of ARF1 in MDA-MB-231 cells preclude further effects of ARF1-Q71L on ERK1/2 phosphorylation. Likewise, treatment with 10 ng/ml ActD or 25 nM VLB resulted in non-significant difference in the levels of phospho-ERK1/2 compared to untreated cells (Fig 6A and 6B, lanes 3, and Fig 6C). In contrast, we found that treatment with ActD or VLB in cells expressing ARF1-Q71L, but not in cells expressing ARF1-T31N, dramatically reduced the levels of phospho-ERK1/2 (Fig 6A and 6B, lanes 4, Fig 6C, and S3 Fig). These reductions in the levels of phospho-ERK1/2 were again consistent with a synergistic effect.

We next analyzed the extent of AKT signaling activation, and we found that the expression of ARF1-Q71L, but not ARF1-T31N, resulted in a significant increase in the levels of phospho-AKT (Fig 6A and 6B, lanes 1 and 2, Fig 6D, and data not shown), which in this case is in agreement with the postulated role of ARF1 in triple-negative breast cancer cells in promoting the activation of survival pathways that include AKT signaling [96]. Treatment with ActD or VLB alone, however, resulted in non-significant changes in the levels of phospho-AKT (Fig 6A and 6B, lanes 3, and Fig 6D), indicating that these drugs at the doses that we used did not trigger survival signaling through ERK1/2 or AKT. In contrast, treatment with each of the antitumor drugs in cells expressing ARF1-Q71L (Fig 6A and 6B, lanes 1 and 4), but not in cells expressing ARF1-T31N (data not shown), showed a significant increase in the levels of phospho-AKT compared to untreated cells (Fig 6D). However, the respective increase was not significant compared to cells expressing ARF1-Q71L only (Fig 6A and 6B, lanes 2 and 4, and Fig 6D), indicating that the combined treatment did not further activate or deactivate AKT. Together, these results indicate that the activation of AKT by ARF1-Q71L was not sufficient to compensate for the inactivation of ERK1/2 by the combined treatments. Thus, it is more likely that ERK1/2 inactivation plays an important role in the effects on cell proliferation, cell migration and apoptosis when MDA-MB-231 cells expressing ARF1-Q71L were treated with ActD or VLB. This could be explained by the early effects of ARF1-Q71L on the secretory pathway, mentioned before, that could result in either impaired transport of signaling receptors to the cell surface and/or the activation of their endocytosis and degradation, especially those involved in ERK1/2 signaling, such as members of the ERBB cell-surface receptor tyrosine kinase family [70, 101]. Likewise, the transport of other secretory pathway cargos could be affected, such as of some ATP binding cassette transporters that likely confer multidrug resistance to MDA-MB-231 cells [102]. In fact, this last possibility could explain the synergistic effects that we have described so far. At the same time, it is possible that receptors involved in AKT signaling are less sensitive to the early effects of ARF1-Q71L on intracellular trafficking, while keeping their sensitivity to ARF1-induced activation.

**Fig 7:**
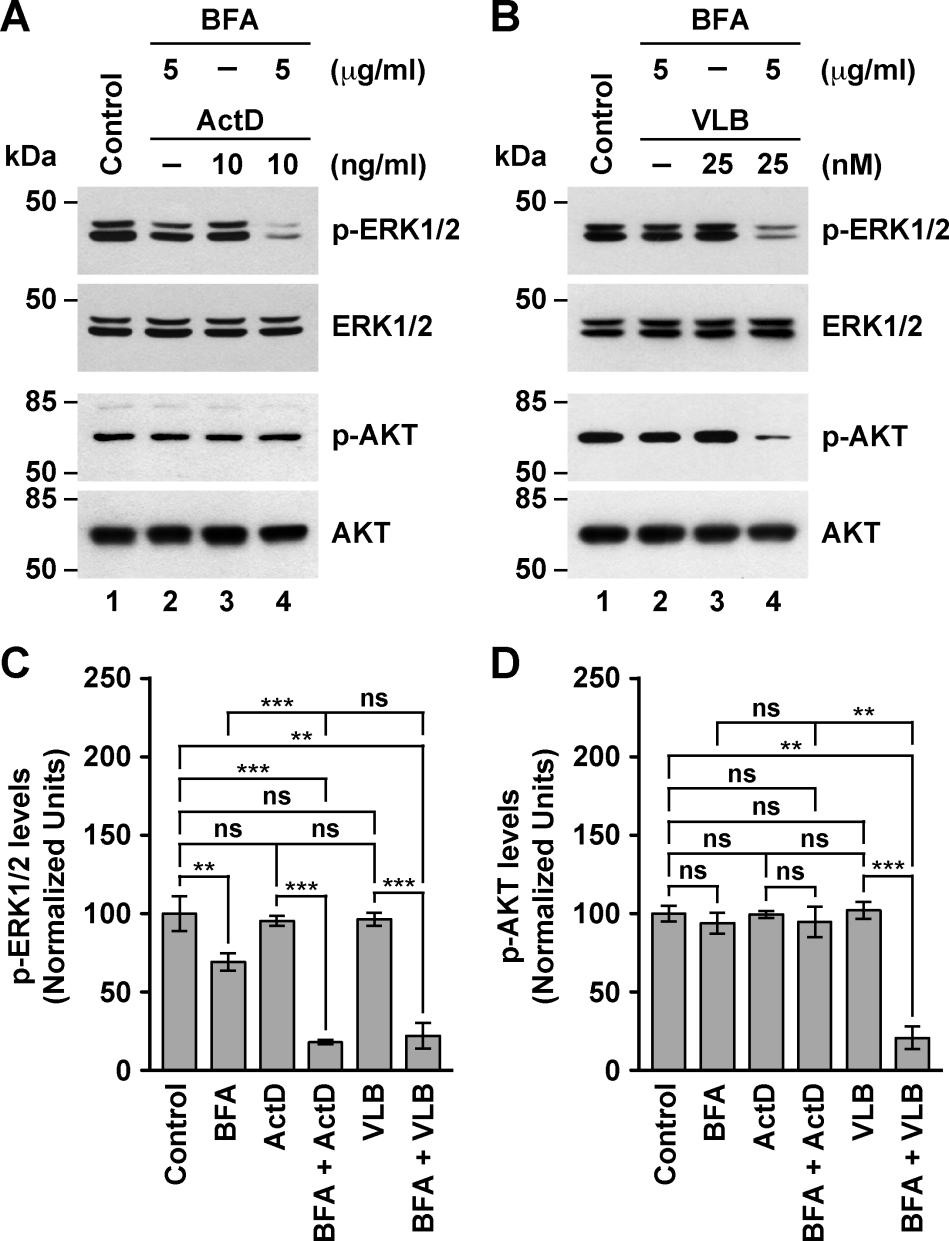
Effect of the combined treatment of Brefeldin A with Actinomycin D or Vinblastine on the levels of phospho-ERK1/2 and phospho-AKT in MDA-MB-231 cells. (A-B) Cells were left untreated for 5 h (*Control*), or treated 5 h either with 5 μg/ml Brefeldin A (*BFA*), 10 ng/ml Actinomycin D (*ActD*; A) or 25 nM Vinblastine (*VLB*; B), or with BFA in conjunction either with ActD (*BFA + ActD*; A) or VLB (*BFA + VLB*; B). After solubilizing in detergent, proteins were analyzed by SDS-PAGE followed by immunoblotting using antibodies to the proteins indicated on the right. The position of molecular mass markers is indicated on the left. (C-D) Densitometry quantification of the immunoblot signal of the levels of phospho-ERK1/2 shown as in *A* and *B* (C), and of the levels of phospho-AKT shown as in *A* and *B* (D). Bar represents the mean ± standard deviation (n = 3). ** *P* < 0.01; *** *P* < 0.001; ns, not statistically significant.

In contrast to the effect of ARF1 mutants, treatment with BFA alone significantly decreased the levels of phospho-ERK1/2 (Fig 7A and 7B, lanes 1 and 2), in a dose-dependent manner (Image A in S4 Fig). This result is consistent with a previous report showing that in rat neurons, treatment with 0.5 μg/ml BFA decreases the levels of phospho-ERK1/2 in a time-dependent manner [103]. Interestingly, the treatment with 5 μg/ml BFA in conjunction with either of the two drugs resulted in a significant decrease in the levels of phospho-ERK1/2 in a manner that was indicative of a synergistic effect (Fig 7A and 7B, lanes 4, and Fig 7C). In contrast, the levels of phospho-AKT were not significantly changed in cells treated with BFA alone (Fig 7A and 7B, lanes 1 and 2, and Fig 7D), or in conjunction with ActD (Fig 7A, lane 4, and Fig 7D). These findings are in contrast to the BFA-induced, time-dependent decrease in the levels of phospho-AKT reported for rat neurons [103, 104], indicating that the AKT signaling in MDA-MB-231 cells is more resistant to BFA, which is also consistent with the levels of ARF1 in these cells [70, 72]. However, treatment of cells with BFA and VLB resulted in a significant decrease in the levels of phospho-AKT (Fig 7B, lane 4, and Fig 7D), suggesting that both compounds act synergistically to affect the AKT pathway in MDA-MB-231 cells. Because microtubules play an important role in membrane trafficking [105], this synergistic effect could be the result of VLB adding an impact on the secretory pathway. The effect of BFA in MDA-MB-231 cells is also in contrast to its effect in differentiated 3T3-L1 adipocytes, which results in an increase in the levels of phospho-AKT [106], or in human keratinocytes, which results in no change in the levels of phospho-AKT [107]. These observations indicate that BFA in different cell types might have several, distinct targets that are related in different fashions to the AKT signaling pathway. In fact, in addition to inhibiting GBF1, which is associated with ARF1-mediated recruitment of COPI at the *cis*-Golgi [108], BFA inhibits BIG1 and BIG2, two ARF-GEFs associated with ARF1-mediated recruitment of clathrin coats at the *trans*-Golgi for post-Golgi membrane trafficking, which include endocytic compartments [109]. Thus, the effects of BFA on ERK1/2 and AKT activation could result from altered trafficking of the corresponding signaling receptors at the Golgi apparatus-cell surface interface. This notion is supported by data showing that treatment of gastric cancer cells with M-COPA, another inhibitor of ARF1 function [32], downregulates cell surface expression of the receptor tyrosine kinases MET and FGFR2, resulting in antitumor activity [110].

### The treatment of MDA-MB-231 cells with ActD in conjunction with GCA affects AKT signaling

**Fig 8:**
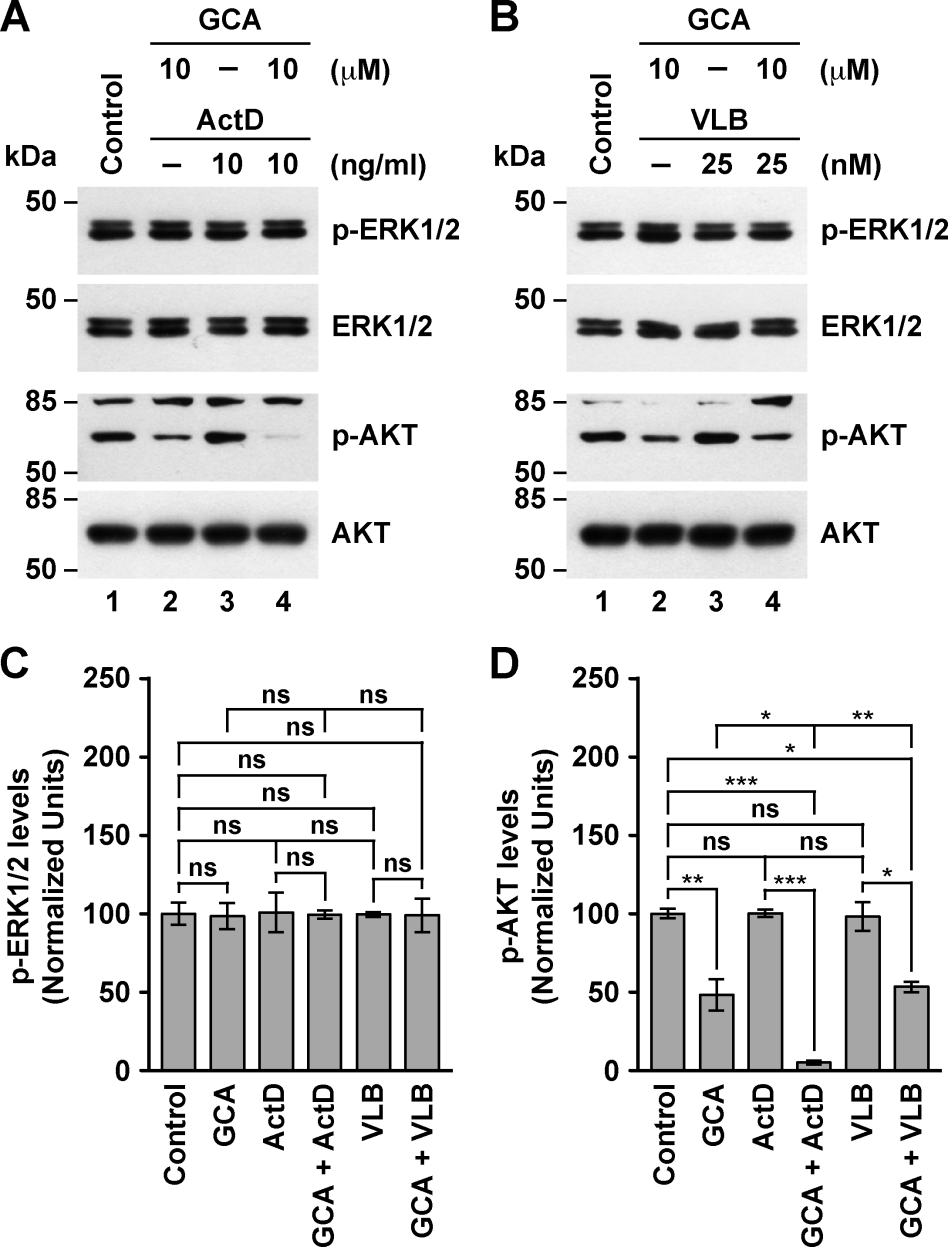
Effect of the combined treatment of Golgicide A with Actinomycin D or Vinblastine on the levels of phospho-ERK1/2 and phospho-AKT in MDA-MB-231 cells. (A-B) Cells were left untreated for 5 h (*Control*), or treated 5 h either with 10 μM Golgicide A (*GCA*), 10 ng/ml Actinomycin D (*ActD*; A) or 25 nM Vinblastine (*VLB*; B), or with GCA in conjunction either with ActD (*GCA + ActD*; A) or VLB (*GCA + VLB*; B). After solubilizing in detergent, proteins were analyzed by SDS-PAGE followed by immunoblotting using antibodies to the proteins indicated on the right. The position of molecular mass markers is indicated on the left. (C-D) Densitometry quantification of the immunoblot signal of the levels of phospho-ERK1/2 as shown in *A* and *B* (C), and of the levels of phospho-AKT as shown in *A* and *B* (D). Bar represents the mean ± standard deviation (n = 3). * *P* < 0.05; ** *P* < 0.01; *** *P* < 0.001; ns, not statistically significant.

Next, we analyzed the effect of the combined treatments with GCA on ERK1/2 and AKT signaling. Surprisingly, we found that treatment with GCA alone, up to 10 μM, had no effect on the levels of phospho-ERK1/2 (Fig 8A and 8B, lanes 1 and 2, and Fig 8C), in contrast to the effect of BFA (Fig 7 and S4 Fig). However, the same treatments with GCA resulted in a significant, dose-dependent decrease in the levels of phospho-AKT (Fig 8A and 8B, lanes 1 and 2, Fig 8D, and S4 Fig), which is also in contrast to the effect of BFA (Fig 7 and S4 Fig). Treatment of ActD in conjunction with GCA also showed no effect on the levels of phospho-ERK1/2 (Fig 8A, lane 4, and Fig 8C). However, the same treatment produced a significant decrease on the levels of phospho-AKT, and to an extent that suggests a synergistic effect (Fig 8A, lane 4, and Fig 8D). In contrast, the combined treatment of GCA and VLB showed no effect on the levels of phospho-ERK1/2 or of phospho-AKT (Fig 8B–8D). These results indicate that although both BFA and GCA target ARF1 function, each might have additional or different targets such that they affect the ERK1/2 and AKT pathways distinctly. As mentioned before, BFA inhibits *cis*-Golgi GBF1 and the *trans*-Golgi BIG1 and BIG2, while GCA seems to be specific for GBF1 [36]. Therefore, the results obtained with GCA are consistent with the notion that its effect on AKT phosphorylation is primarily via mechanisms at the endoplasmic reticulum-Golgi apparatus interface. Instead, the effect of BFA on ERK phosphorylation is consistent with an effect on post-Golgi mechanisms regulating the activation of ERK signaling. It will be important to determine whether ERK1/2 and AKT respond equally to BFA and GCA in other triple-negative breast cancer cells. Similarly, the synergistic effect of VLB could then be the result of more sensitive microtubule dynamic mechanisms of *trans*-Golgi or post-Golgi membrane trafficking processes. On the other hand, the synergistic effect of GCA and ActD on AKT phosphorylation suggests the intriguing possibility that ActD affects mechanisms that connect the early secretory pathway with gene transcription, such as some that are activated during stress responses due to overload of secretory pathway cargos [111].

### Concluding remarks

The MDA-MB-231 cell line has been extensively used as a model of triple-negative breast cancer, which is an aggressive subgroup of human breast cancer characterized by the lack of expression of estrogen receptor and progesterone receptor, and lack of overexpression of human epidermal growth factor receptor 2. It accounts for close to 15% of all types of breast cancer [112]. Importantly, triple-negative breast cancer is an intrinsically heterogeneous disease with diverse physiological and pathological features associated with the aggressive phenotype [43]. It is the most difficult subgroup to treat, due to its unresponsiveness to currently available receptor-targeted therapies, leaving these cancers with limited treatment options [98]. For these reasons, a large number of new therapeutic strategies are under broad investigation [113]. The results presented in our report suggest that disruption of Golgi function could be used as a new strategy for the sensitization to chemotherapy of this kind of cancer. Thus, it will be important to validate this possibility in other triple-negative breast cancer cell lines, as well as on cells from triple-negative breast cancer tissue. In addition, our results suggest that different combinations of Golgi disruptors and antitumor drugs could be used to selectively target transforming pathways, with deleterious effects on the tumorigenic phenotype. In this regard, inhibition of ARF1 function has been tested as potential therapeutic target for cancer, including triple-negative breast cancer [32, 70, 72, 83, 96, 114, 115]. However, our results indicate a more complex relationship than anticipated between ARF1 function and the targets of ARF-GEFs inhibitors that could be exploited therapeutically. On the one hand, short-term expression of ARF1-Q71L revealed a possible relevant sensitivity of the ERK signaling pathway in cells overexpressing ARF1, and on the other hand, BFA and GCA, with their inhibiting effects on ARF1 function, showed distinct sensitizations to the anticancer drugs ActD and VLB.

## Supporting information

S1 Fig Effect of the combined treatments of ARF1 disruptors and antitumor drugs on the Golgi apparatus of MDA-MB-231 cells. Cells were left untreated (A; *Control*), or transfected to transiently express the HA-epitope-tagged ARF1 constitutively-activated mutant, and after 16-h further treated for 1-h either with 10 ng/ml Actinomycin D (B; *HA-ARF1-Q71L + ActD*) or 25 nM Vinblastine (C; *HA-ARF1-Q71L + VLB*). Other cells were treated for 1-h either with 5 μg/ml Brefeldin A (D and E) or 10 μM Golgicide A (F and G) in conjunction either with 10 ng/ml Actinomycin D (D: *BFA + ActD*; F: *GCA + ActD*) or 25 nM Vinblastine (E: *BFA + VLB*; G: *GCA + VLB*). Cells were fixed, permeabilized, and immunolabeled with mouse monoclonal antibody to GM130, rabbit polyclonal antibody to Giantin, and sheep antibody to TGN46. Secondary antibodies were Alexa-594-conjugated donkey anti-mouse IgG (red channel), Alexa-488-conjugated donkey anti-rabbit IgG (green channel), and Alexa-647-conjugated donkey anti-sheep IgG (blue channel). Nuclei were stained with DAPI (gray channel). Stained cells were examined by fluorescence microscopy. Merging red, green, blue, and grey channels generated the fourth image on each row; yellow indicates overlapping localization of the red and green channels, cyan indicates overlapping localization of the green and blue channels, magenta indicates overlapping localization of the red and blue channels, and white indicates overlapping localization of all three channels. Bar, 10 μm.
(TIF)

S2 Fig Dose-response effects of the treatments with Actinomycin D, Vinblastine, or Golgi disrupting agents on the proliferation of MDA-MB-231 cells. Cells were left in normal culture medium containing 10% FBS (A-D), or transfected with the indicated concentrations of plasmid DNA to transiently express for 16 h the HA-epitope-tagged ARF1 constitutively-activated mutant (E). Untransfected cells were either maintained for 24 h in normal culture medium containing 10% FBS (*Serum*), or serum-starved, and either left untreated for additional 24 h (*Control*) or treated 24 h with the indicated concentrations of either Actinomycin D (*ActD*; A), Vinblastine (*VLB*; B), Brefeldin A (*BFA*; C) or Golgicide A (*GCA*; D). Transfected cells were serum-starved and left without further treatment for additional 24 h (E). In all conditions, cells were cultured during the last 24 h in the presence of [^3^H]-thymidine. Cells were harvested, and [^3^H]-thymidine incorporation was quantified with a scintillation counter. Bar represents the mean ± standard deviation (n = 3). * *P* < 0.05; ** *P* < 0.01; *** *P* < 0.001; ns, not statistically significant.
(TIF)

S3 Fig Effect of the expression of ARF1 variants alone or in conjunction with the treatment with Actinomycin D or Vinblastine on the levels of phospho-ERK1/2 in MDA-MB-231 cells. (A-B) Cells were left untreated (*Control*), or transfected to transiently express for 16 h either the HA-epitope-tagged ARF1 dominant-negative mutant (*ARF1-T31N*) or the constitutively-activated mutant (*ARF1-Q71L*). Cells were left untreated for further 5 h (*Control*, *ARF1-T31N* and *ARF1-Q71L*; A and B), or treated 5 h either with 10 ng/ml Actinomycin D (*ActD*; A) or 25 nM Vinblastine (*VLB*; B). After solubilizing in detergent, proteins were analyzed by SDS-PAGE followed by immunoblotting using antibodies to the proteins indicated on the right, or to the HA-epitope to detect ARF1 variants. The position of molecular mass markers is indicated on the left. (C) Densitometry quantification of the immunoblot signal of the levels of phospho-ERK1/2 as shown in *A* and *B*. Bar represents the mean ± standard deviation (n = 3). *** *P* < 0.001; ns, not statistically significant.
(TIF)

S4 Fig Dose-response effect of Brefeldin A or Golgicide A on the levels of phospho-ERK1/2 and phospho-AKT. (A-B) Cells were left untreated for 5 h (*Control*; A and B), or treated 5 h with the indicated concentrations of Brefeldin A (*BFA*; A), or the indicated concentrations of Golgicide A (*GCA*; B). After solubilizing in detergent, proteins were analyzed by SDS-PAGE followed by immunoblotting using antibodies to the proteins indicated on the right. The position of molecular mass markers is indicated on the left.
(TIF)
