# Supplementary figures and images for: Functional disruption of the Golgi apparatus protein ARF1 sensitizes MDA-MB-231 breast cancer cells to the antitumor drugs Actinomycin D and Vinblastine through ERK and AKT signaling
Source: PLoS One. 2018 Apr 3;13(4):e0195401. doi: 10.1371/journal.pone.0195401 (PMC5882166; doi:10.1371/journal.pone.0195401)

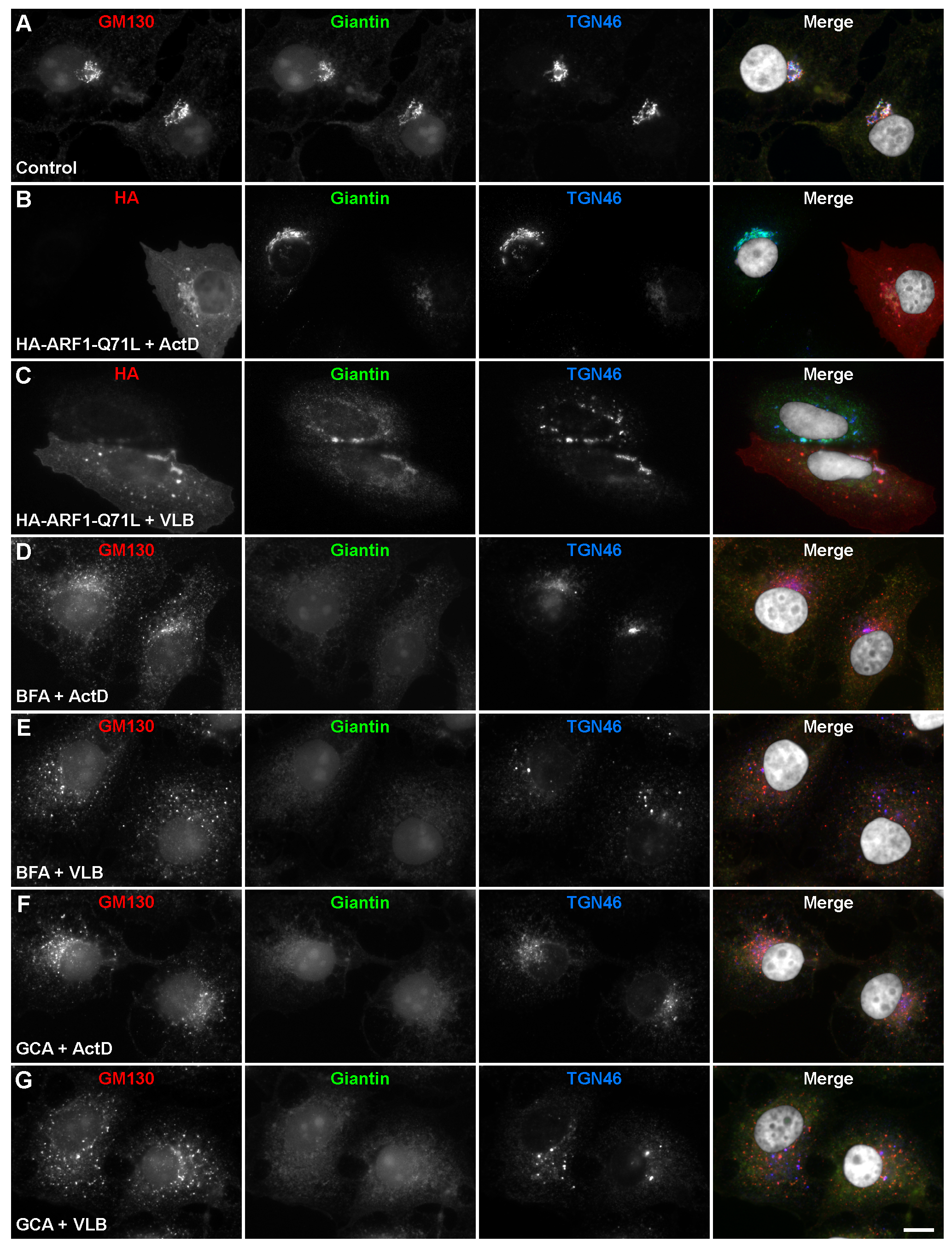

Supplement: S1 Fig — Cells were left untreated (A; Control), or transfected to transiently express the HA-epitope-tagged ARF1 constitutively-activated mutant, and after 16-h further treated for 1-h either with 10 ng/ml Actinomycin D (B; HA-ARF1-Q71L + ActD) or 25 nM Vinblastine (C; HA-ARF1-Q71L + VLB). Other cells were treated for 1-h either with 5 μg/ml Brefeldin A (D and E) or 10 μM Golgicide A (F and G) in conjunction either with 10 ng/ml Actinomycin D (D: BFA + ActD; F: GCA + ActD) or 25 nM Vinblastine (E: BFA + VLB; G: GCA + VLB). Cells were fixed, permeabilized, and immunolabeled with mouse monoclonal antibody to GM130, rabbit polyclonal antibody to Giantin, and sheep antibody to TGN46. Secondary antibodies were Alexa-594-conjugated donkey anti-mouse IgG (red channel), Alexa-488-conjugated donkey anti-rabbit IgG (green channel), and Alexa-647-conjugated donkey anti-sheep IgG (blue channel). Nuclei were stained with DAPI (gray channel). Stained cells were examined by fluorescence microscopy. Merging red, green, blue, and grey channels generated the fourth image on each row; yellow indicates overlapping localization of the red and green channels, cyan indicates overlapping localization of the green and blue channels, magenta indicates overlapping localization of the red and blue channels, and white indicates overlapping localization of all three channels. Bar, 10 μm. (TIF) [file pone.0195401.s001.tif]

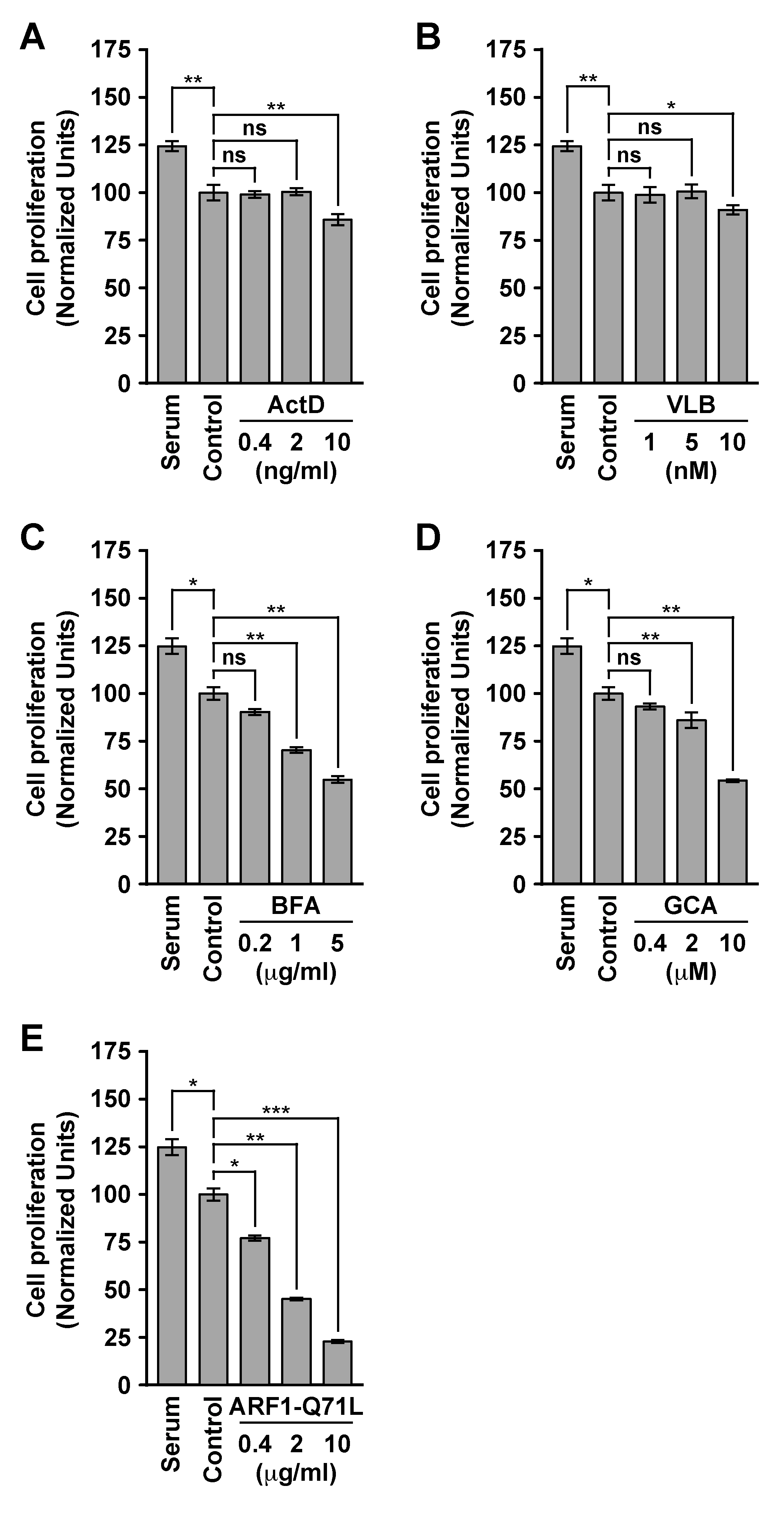

Supplement: S2 Fig — Cells were left in normal culture medium containing 10% FBS (A-D), or transfected with the indicated concentrations of plasmid DNA to transiently express for 16 h the HA-epitope-tagged ARF1 constitutively-activated mutant (E). Untransfected cells were either maintained for 24 h in normal culture medium containing 10% FBS (Serum), or serum-starved, and either left untreated for additional 24 h (Control) or treated 24 h with the indicated concentrations of either Actinomycin D (ActD; A), Vinblastine (VLB; B), Brefeldin A (BFA; C) or Golgicide A (GCA; D). Transfected cells were serum-starved and left without further treatment for additional 24 h (E). In all conditions, cells were cultured during the last 24 h in the presence of [3H]-thymidine. Cells were harvested, and [3H]-thymidine incorporation was quantified with a scintillation counter. Bar represents the mean ± standard deviation (n = 3). * P < 0.05; ** P < 0.01; *** P < 0.001; ns, not statistically significant. (TIF) [file pone.0195401.s002.tif]

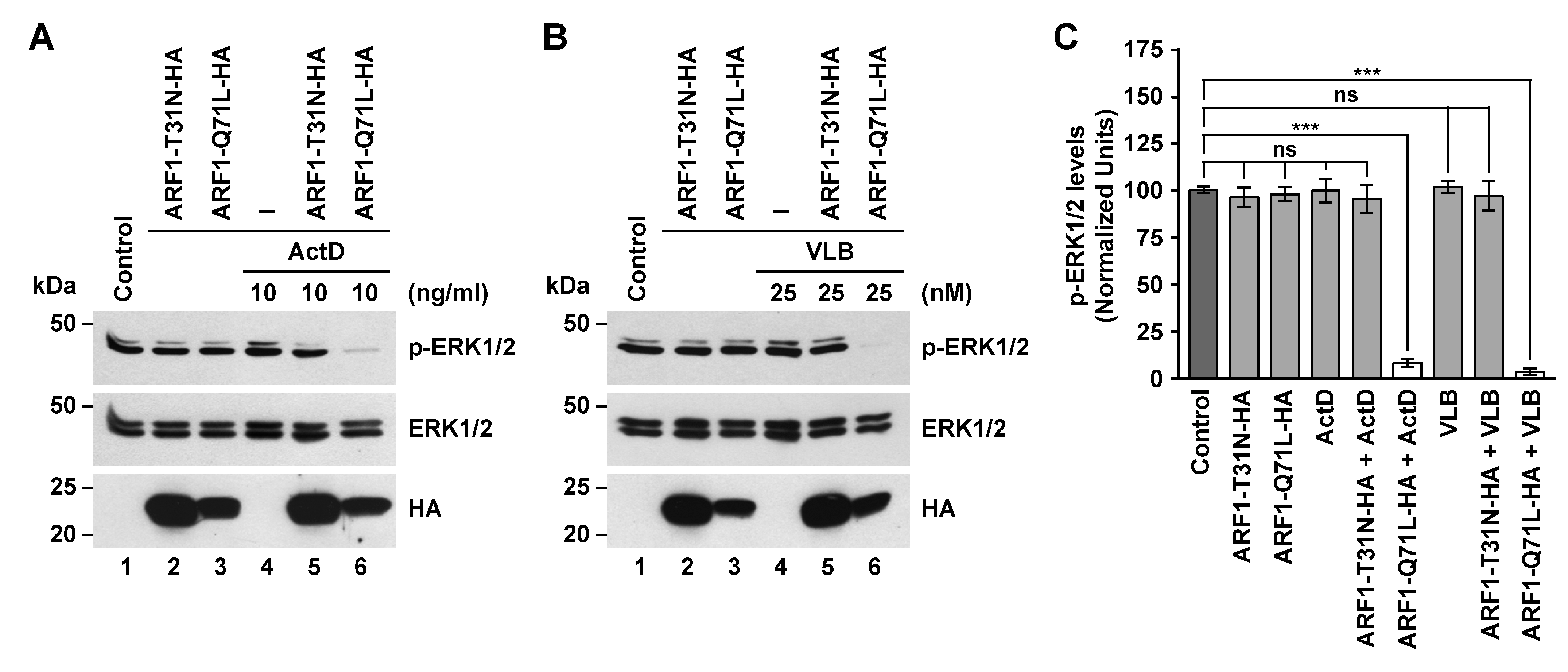

Supplement: S3 Fig — (A-B) Cells were left untreated (Control), or transfected to transiently express for 16 h either the HA-epitope-tagged ARF1 dominant-negative mutant (ARF1-T31N) or the constitutively-activated mutant (ARF1-Q71L). Cells were left untreated for further 5 h (Control, ARF1-T31N and ARF1-Q71L; A and B), or treated 5 h either with 10 ng/ml Actinomycin D (ActD; A) or 25 nM Vinblastine (VLB; B). After solubilizing in detergent, proteins were analyzed by SDS-PAGE followed by immunoblotting using antibodies to the proteins indicated on the right, or to the HA-epitope to detect ARF1 variants. The position of molecular mass markers is indicated on the left. (C) Densitometry quantification of the immunoblot signal of the levels of phospho-ERK1/2 as shown in A and B. Bar represents the mean ± standard deviation (n = 3). *** P < 0.001; ns, not statistically significant. (TIF) [file pone.0195401.s003.tif]

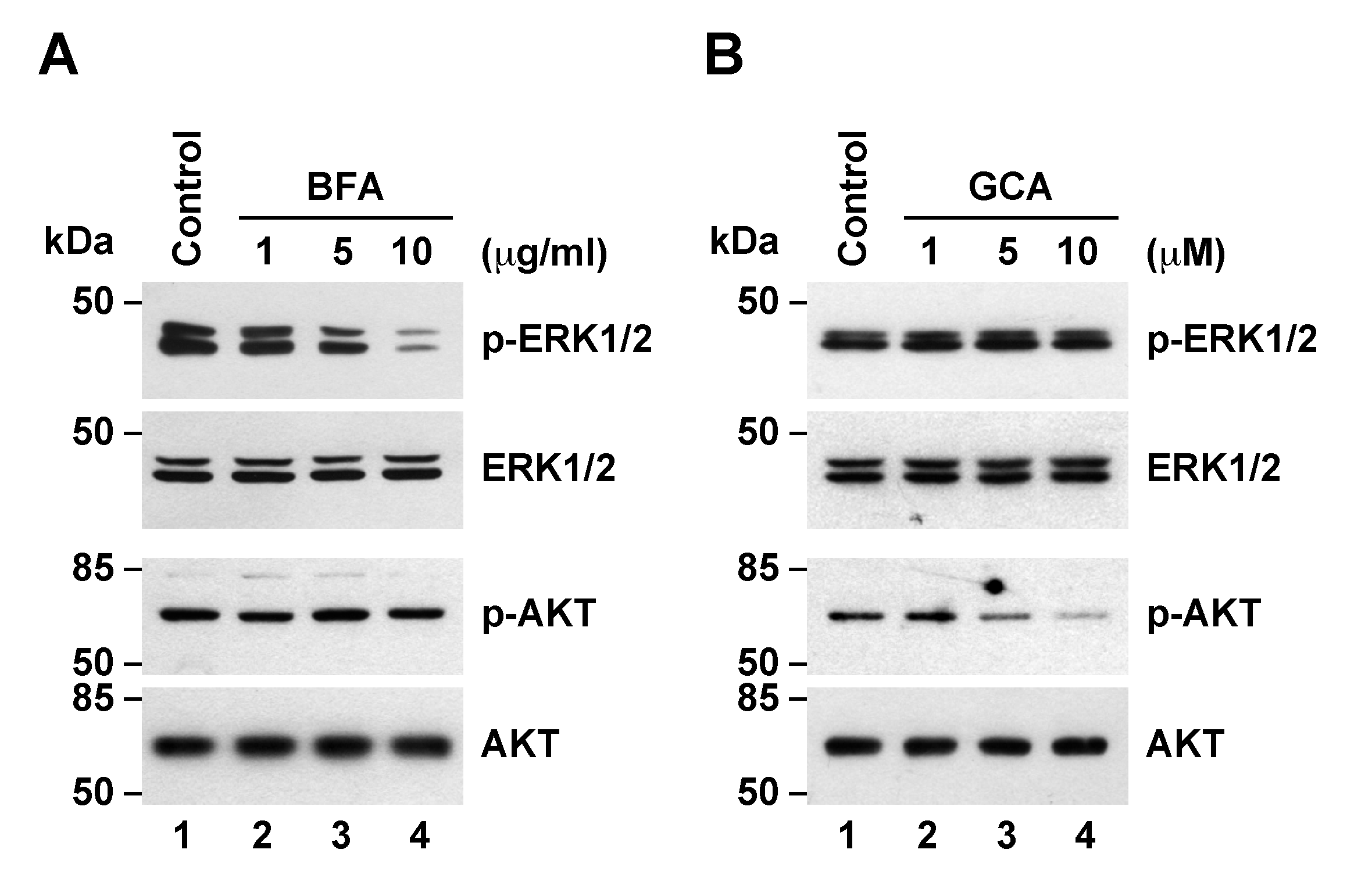

Supplement: S4 Fig — (A-B) Cells were left untreated for 5 h (Control; A and B), or treated 5 h with the indicated concentrations of Brefeldin A (BFA; A), or the indicated concentrations of Golgicide A (GCA; B). After solubilizing in detergent, proteins were analyzed by SDS-PAGE followed by immunoblotting using antibodies to the proteins indicated on the right. The position of molecular mass markers is indicated on the left. (TIF) [file pone.0195401.s004.tif]
